# 1197. Pharmacist-assisted urine culture review and antimicrobial stewardship interventions in the emergency department: A study from the Middle East Gulf region

**DOI:** 10.1093/ofid/ofad500.1037

**Published:** 2023-11-27

**Authors:** Mohamed Hisham, Rania ElLababidi, Fatima Alsalama, Hazem Elrefaei, Rama Nasef, Muhammad Zeeshan Khan, Timothy Souster

**Affiliations:** Cleveland Clinic Abu Dhabi, Abu Dhabi, Abu Dhabi, United Arab Emirates; Cleveland Clinic Abu Dhabi, Abu Dhabi, Abu Dhabi, United Arab Emirates; Cleveland Clinic Abu Dhabi, Abu Dhabi, Abu Dhabi, United Arab Emirates; Cleveland Clinic Abu Dhabi, Abu Dhabi, Abu Dhabi, United Arab Emirates; Cleveland Clinic Abu Dhabi, Abu Dhabi, Abu Dhabi, United Arab Emirates; Cleveland Clinic Abu Dhabi, Abu Dhabi, Abu Dhabi, United Arab Emirates; Cleveland Clinic Abu Dhabi, Abu Dhabi, Abu Dhabi, United Arab Emirates

## Abstract

**Background:**

Urinary tract infection (UTI) is one of the most frequent reasons encountered for emergency department (ED) visits. The treatment of asymptomatic bacteriuria (ASB) is identified as one of the causes of inappropriate antimicrobial use. We designed the study to evaluate and improve the prescribing practices for UTIs in Cleveland Clinic Abu Dhabi ED. To our knowledge, this is one of the first studies in the Middle East Gulf region which evaluated the impact of pharmacist-assisted urine culture review in the ED.

**Methods:**

A pre-post quasi-experimental study was conducted between September 2021 and July 2022 with a 4-month pre-intervention phase, a 3- month wash-out period, and a 4-month post-intervention phase. The pharmacist-assisted antimicrobial stewardship interventions included distinguishing between UTI and ASB, proper screening through diagnostic stewardship, and rationalizing the need to order urine cultures. The major interventions included appropriate antimicrobial therapy, dosing, frequency, and duration of therapy in compliance with the hospital guidelines. All patients with a negative urine culture, aged ≤ 18 years, or requiring inpatient admission during the same ED visit were excluded from the study. Data were analyzed using Fisher’s exact test.

**Results:**

Three hundred and seventy ED patients were included in the study. Thirty percent of the urine cultures were ordered for patients without urinary signs and symptoms. *Escherichia coli* was the predominant urinary isolate in our study from both groups, followed by *Klebsiella* species and *Streptococcus agalactiae*. Antimicrobials were prescribed for asymptomatic bacteriuria in 5.2 % of patients in the post-intervention group and 21.8 % in the pre-intervention group (*P* = 0.0001). Readmission to ED for the same complaints within 30 days of the ED visit occurred in 9.8 % of patients in the post-intervention group and 13.2 % in the pre-intervention group (*P* = 0.3337). All study findings are presented in the tables.

**Table 1:**
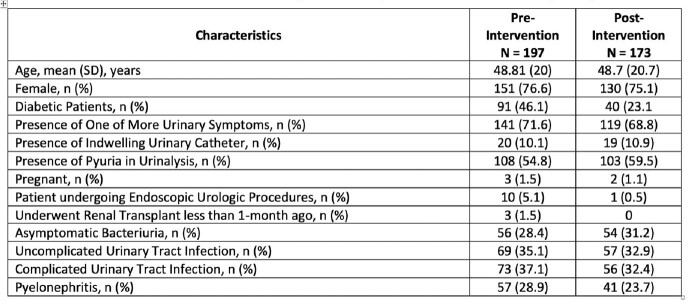
Characteristics of the pre-and post-intervention group

**Table 2:**
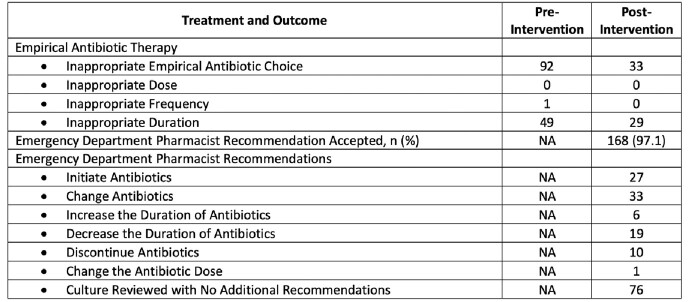
Treatment and outcome of the pre-and post-intervention group

**Table 3:**
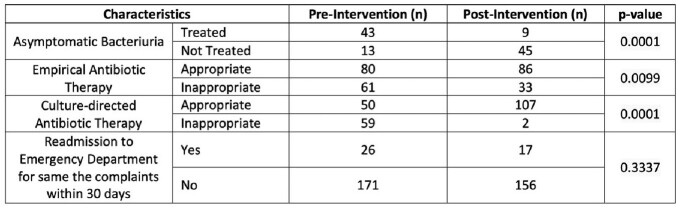
Impact of pharmacist-assisted urine culture review in the emergency department

**Conclusion:**

Our study observed higher rates of appropriate culture-direct antimicrobial therapy after implementing the pharmacist-assisted urine culture review and positively impacted clinical outcomes. Additionally, there was a significant reduction in treating asymptomatic bacteriuria.

**Disclosures:**

**All Authors**: No reported disclosures

